# Epidemiology and clinical manifestation of fungal infection related to Mucormycosis in hematologic malignancies

**Published:** 2015

**Authors:** M Noorifard, E Sekhavati, H Jalaei Khoo, I Hazraty, R Tabrizi

**Affiliations:** * Department of Infectious Diseases, AJA University of Medical Sciences, Tehran, Iran; ** Larestan School of Medical Sciences, Larestan, Iran; *** Department of Hematology & Oncology, AJA University of Medical Sciences, Tehran, Iran; **** Department of Anesthesiology and Critical Care, AJA University of Medical Sciences, Tehran, Iran; ***** Health Policy Research Center, Shiraz University of Medical Sciences, Shiraz, Iran

**Keywords:** epidemiology, Mucormycosis, hematologic malignancy

## Abstract

**Introduction:** Mucormycosis is an opportunist fungus infection with acute and rapidly progressive nature in the hematologic malignancy patients. This study was done to investigate the prevalence and clinical manifestations of this infection among hematologic malignancies.

**Methodology:**This cross-sectional study (descriptive-analytical) was performed while investigating medical records of 30 patients with hematologic malignancy affected by Mucormycosis in Imam Reza Hospital between 2001 and 2013. After collecting the data, it was entered in SPSS 19 Software with a provided checklist that included demographic characteristics, clinical manifestations, and it was analyzed by using descriptive (mean, frequency) and inferential (chi- square and independent -t-test) statistical methods (p-value < 0.05 was considered as statistically significant).

**Findings:**Overall, the prevalence of Mucormycosis was 4.29 per 100 patient hematologic malignancies. The infection proportion among men and women was 72. 2, 27.6%, respectively. The maximum cases of Mucormycosis were observed among AML patients (62.1%). The most common place of involvement was lung (89.4%) and fever was the most popular sign of the infection (100%). The most considerable and effective factor in the prognosis of infection was using combined therapy of Amphotericin Band surgery (debridement) that has statistically significant correlation (p<0.05).

**Conclusion:**Considerable prevalence and death related to Mucormycosis infection among patients of hematologic malignancy showed the importance of having strategies for its prevention and early diagnosis especially among acute leukemia patients.

## Introduction

Mucormycosis is one of original order of fungus Zygomycetes that is considered as important human pathogens. Mucormycosis is a fatal, opportunist fungus with acute infectious and rapid progress nature. Although this aggressive fungal infection is rare, it is common among patients whose immune system of their body has been disturbed for different reasons [**21**]. 

During recent decades, a high percent of infected patients by this infection has been observed significantly among diabetic patients, malnutrition children, patients with severe burning, people affected by hematologic malignancy and during therapy with immunosuppressive, cytotoxins, corticosteroids or after surgery and trauma [**2**,**12**,**15**]. In patients with hematologic malignancies, this fungus causes death by its effect and delays or stops the influence of anticancer treatment. Among common forms of Mucormycosis disease, we can mention pulmonary, rhino-cerebral, gastrointestinal, cutaneous, and distributed form [**8**]. Mucormycosis causes emboli and necrosis of the tissues by invading blood vessels and spreads through the body by blood current [**7**,**8**]. Generally, the infection prognosis depends on several factors including infection place, speed of disease diagnosis, type of cancer, extension, and severity based upon the fungus’ invasion and host body’s immune system [**20**]. The early diagnosis of the disease is very important from the treatment view. Unfortunately, doubt is focused on clinical symptoms of the disease. Also, diagnosis probability of the disease before death is just 23- 50%. Early medical and surgery treatment can prevent Mucormycosis development and in some cases, it may remove the disease with surgery (depending on expansion and lesion) and Amphotericin B [**10**,**11**].

During the two last decades, the studies have shown an increase of Mucormycosis compared to other invading fungi as Aspergillus among people having deficiency in immune system [**17**]. In 2002, the study of Marr et al. showed that the number of Mucormycosis patients has increased more than two-fold between 1985- 9 and 1995- 9 [**14**]. Based on a study in America, the annual incidence rate of Mucormycosis was of 1.7 cases per 1 million people in the general population or in other words, the average was of approximately 500 American people per year [**9**]. Since a very low rate of this infection is recognizable before death, these figures were probably underestimated. Also a number of these cases are not recognizable due to autopsy reduction in America and Europe (from 60% in 1960 to 10% at present) [**11**,**17**].

At present, the prevalence of this infection among patients with deficiency in immune system in Imam Reza Hospital has increased according to the infectious experts’ opinions. Since there are no adequate studies about the prevalence and relationship between hematologic malignancy and Mucormycosis [**21**], the present study’s aim was to investigate the frequency of the infection and the probable factors causing Mucormycosis among hematologic malignancies in Imam Reza Hospital.

## Methodology

The present study is a descriptive-analytical study of retrospective cross-sectional type and was carried out for blood units of men and women hospitalized in Imam Reza Hospital of Tehran between 2001 and 2013, after getting the permission and the required coordination. This study was limited to adult patients (> 15 years old) with hematologic malignancies (AML, ALL, CML, CLL, NHL, HL (HD), MM (multiple myeloma)) and patients with different kinds of hematologic malignancies like myelodysplastic syndromes or non-malignant blood disorders (aplastic anemia, thrombocytopenia and hemolytic anemia, etc.), who were excluded from the study. A checklist including age, gender, disease prognosis, treatment method, season of occurrence, place of involvement infection, concurrent involvement of the lung or being diabetic, job, Para-clinical tests (like WBC, BUN, FBS, HCO3, PH), type of malignancy, beginning time of symptoms and initiation of treatment, previous hospitalization history, way of diagnosis and signs and symptoms of the disease based on the existing medical records and evidence in medicine and hematology units of the hospital, were prepared for each patient of hematologic malignancy in whom Mucormycosis infection was diagnosed. In this study, Mucormycosis infection diagnosis was performed according to clinical manifestations evaluation of the disease from views of experts in infection, hematology, ear, nose and throat (ENT) specialists or included details investigation based on history and otorhinolaryngological examination (ear, nose and throat), eyes, lung and heart examination, abdomen, neurology and, if required, experimental investigation or microbiology based on histopathology or prepared culture from biopsy of nasal cavity, sinuses and roof of the mouth, lung, heart or necrosis tissue and radiographic images (like CT, MRI, etc.). Data was entered in SPSS 19 Software after collecting and analyzing it by using descriptive and inferential statistical methods (mean, frequency, independent –t, chi-square, and exact test of Fisher) and p-value < 0.05 was considered as statistically significant.

## Results

According to the results of the study, 29 cases of Mucormycosis infection were recognized in 677 patients with hematologic malignancy. Generally, the prevalence of the disease was 4.29 per 100 patients with hematologic malignancy during the studied years. Men with frequency of 21 (72.2%) and mean age of 48 years old (with a range of 20-87 years old) proved to be the maximum cases of infection. Demographic and clinical characteristics of these patients have been summarized in **Table 1**.

**Table 1:** Demographic and clinical characteristics of infected patients

Characteristics	patients (29)
Age (year)	48.48 ± 19.93
Gender (Male / Female)	21/ 8 (72.2/ 27.6)
Job	
Military	10 (34.5)
Civilian	19 (65.5)
History of hospitalization for a reason other than the underlying disease	
Yes	4 (13.8)
No	25 (86.2)
Diabetic history in addition to the underlying disease	
Yes	6 (20.7)
No	23 (79.3)
Season of infection happening	
Spring	9 (31)
Summer	4 (13.8)
Autumn	10 (34.5)
Winter	6 (20.7)
Patient being Neutropenia	
Yes	26 (82.8)
No	3 (17.2)
Chemotherapy experience	
Yes	27 (93.1)
No	2 (6.9)
Type of the underlying malignancy	
AML	18 (62.1)
ALL	6 (20.7)
NHL	2 (6.9)
HCL	1 (3.4)
CLL	1 (3.4)
MM	1 (3.4)
Place of involvement of infection	
Pulmonary (10 is shared with others)	26(89.4)
Orbito-sinus-facial (6 is shared with others)	9 (30.1)
Central Nervous System	6 (20.6)
Gastro-intestinal tract	1 (3.4)
Myocardium	2 (6.8)
*• Patients with Mucormycosis infection.*	
*• Data are Mean ± SD, and Number (%).*	

Most of the patients with Mucormycosis infection had civilian jobs (employee, self-employment, and housekeeping) and the hospitalization was due to a reason other than the underlying disease only in 4 patients. In addition, patients having this infection were diabetic in 20.7% [**20**] in addition to hematologic malignancies. For season of infection happening, autumn included about a third of the Mucormycosis cases.

The results showed that 82.8% of the infections were among acute leukemia patients, of which, 18 cases (62.1%) and 6 cases (20.7%) were influenced respectively by acute myelogenous leukemia (AML) and acute lymphatic leukemia (ALL). Also, all patients except for two persons have experienced chemotherapy previously (average frequency of chemotherapy was of about 4 periods with a range of 1-7 periods).

Based on the results, when the clinical diagnosis of fungal infection was done, 25 patients out of 29 were neutropenia and also, the time interval between the beginning of neutropenia and infection diagnosis was estimated at about 13.1 days (range 5-58 days), and, time interval between the beginning of clinical symptoms and treatment was observed in about 10.65 days (1-55 days

In 26 patients (89.4%) with infection, the lung was the main place of involvement, being the only place of the infection in 16 patients. However, there was also a secondary place in 10 cases in addition to the lung. Other places of the infection included orbit-sinus-facial place in 9 patients (30.1%), CNS in six persons, digestion, and heart system problems in one patient and for each of these places, there were usually secondary places. In addition, the investigation of signs and symptoms of diagnosed patients, based on infection place showed that fever (100% of the patients) was most common sign of systemic infection. As it was shown in **Table 1**, in patients with pulmonary Mucormycosis, orbit-sinus-facial, and CNS respectively; coughing, facial edema, and headache were common symptoms of the infection.

**Table 2:** Signs and symptoms of patients diagnosed based on the place of Mucormycosis infection

Signs and symptoms	patients (29)
Systemic	
Fever	29 (100)
Pulmonary 26 (89.4)	
Cough	22 (84.6)
Dyspnea	20 (76.9)
Chest pain	11 (42.3)
Hemoptysis	2 (7.7)
Orbito-sinus-facial localization (sins-nose-eye) 9 (30.1)	
Facial edema	9 (100)
Nasal obstruction	7 (77.8)
Facial pain	6 (66.7)
Rhinorrhea	5 (55.6)
Eye pain (chemosis- Proptosis- Epiphora)	5 (55.6)
Palate (ulcer destruction)	2 (22.2)
Central nervous system involvement 6 (20.6)	
Headache	4 (66.7)
Facial nerve palsy	2 (33.3)
Hemiplegia	1 (16.7)
Ptosis. Diplopia	1 (16.7)
Myocardium 3 (6.9)	
Dyspnea	1
Thoracic pain	1
Tachycardia	1
Gastro-intestinal tract 2 (3.4)	
Abdominal pain	1
Diarrhea	1
*• Patients with Mucormycosis infection.*	
*• Data are Number (%).*	

The laboratory findings of patients having infection showed that the average WBC count was 18.29 (×103/ μlit) with a range of 0.1-2.10 (×103/ μlit) and number of WBC in 51.7% of the patients was lower than 3.5×103/ μlit while its number in 27.6% of the patients was higher than 10×103/ μlit. Also the average PH of the patients’ serum was 7.38 (range 6-7.6). It was higher than 7.45 among 34.5% of the patients while in 13.8% of the patients it was lower than 7.35. The average of serum bicarbonate was 25.62 mg/ dlit (with range 7-113). This value was more than 29 mg/ dlit in 37.9% of the patients while among 17.2% of the patients it was less than 15 mg/ dlit. In this study, the average value of glucose in the blood, Creatinine of the serum and BUN in the patients having infection were 138.65 (with range of 89-298), 1.10 (0.50-1.90) and 17.27 mg/ dlit, respectively.

In total, of 29 patients having infection, 19 (65.5%) died and 10 (34.5%) survived during the investigation. Also, the analysis showed that although the death rate among women, older, diabetic, neutropenia patients, the ones having civilian jobs and AML underlying disease higher, none of these variables had a significant relation with prognoses of the disease (p>0.05).

All the patients quickly received treatment with Amphotericin B (with dose 1-1.5 mg/kg/day). In addition, surgery was performed in about 5 (17.3%) cases. Also Amphotericin B was replaced by Liposomal Amphotericin B in 2 (6.9%), the patients following the treatment. Based on the kind of treatment, about 72.7% [6] of the patients received treatment with Amphotericin B and 2 of the patients who were treated with Liposomal Amphotericin B died during the study. Moreover, out of the patients who received both Amphotericin B and surgery (debridement) only one person died. So, the number of patients who were alive after being treated with Amphotericin B and surgery (debridement) was higher than the other methods according to the results and this relationship was statistically significant (P <0.05).

## Discussion

In recent decades, Mucormycosis infections among the patients with weakened immune system were increasing [**1**,**18**,**23**] especially those with hematologic deficiency [**6**,**11**,**19**]. In the present study, the prevalence rate of Mucormycosis infection was generally 4.29 per 100 patients of hematologic malignancies and this value is similar with the value in the study report of Sarvestani et al. (4.27 per 100 leukemic patients) [**21**]. The average of age and proportion of men and women having the infection in this study were consonant to other studies [**21**,**24**]. There are different opinions about the influence of age and gender on Mucormycosis infection but in the present study, just like in a recent study of Jagarlamundi et al, male gender was more predominant and it seems that the male gender is a susceptible factor for causing infection [**13**].

Similar to other studies, in this study, the most common blood malignancy was affected by acute leukemic infection like AML followed by ALL [**19**,**21**,**24**]. Therefore, patients with AML are probably at the highest risk of Mucormycosis infection [**5**].

According to the results of this study, the main places of involvement in the infection were orderly lung, paranasal sinuses (face and nose), nervous systems, heart and gastrointestinal tract and similar to results of other studies, the most common place for the Mucormycosis infection among patients with hematologic malignancies being proposed was lung involvement with most common systemic sign i.e. fever [**6**,**11**,**21**].

Since our study, other studies showed that most of the patients experienced chemotherapy and neutropenia [**19**,**24**]. Probably deep and prolonged neutropenia due to cytotoxic therapies (chemotherapy) is the main reason for this infection in underlying hematologic malignancies. This issue supports the importance of neutropenia as a risk factor of causing Mucormycosis infection [**21**]. Then, the recovery of neutrophils [**24**] as well as neutropenia patients’ exposure reduction to the environment may be effective in preventing the infection [**19**,**24**].

Mucormycosis cases have been reported all over the world and in the whole nature. However, their accurate ecology (environment) is not clear [**5**]. In a study performed by Zionistic regime, among 19 Mucormycosis rhino-Orbito-cerebral patients, 16 cases happened during autumn and in another study in Japan, 6 hematologic patients of 7 cases were affected by pulmonary Mucormycosis with a similar seasonal trend during August and September [**16**,**22**]. In our study, autumn included the maximum rate of the infection. Probably, heat transfer system in the therapeutic centers is most important factor in spreading fungal spoors among the patients because of temperature fall in this season. The prevalence of Mucormycosis was reduced significantly in the study of Sarvestani et al. by doing some practices to control and improve the environment condition that included transferring the flowers and live plants outside the units, isolation of neutropenia patients being at risk, wearing masks and controlling air quality for the exposure reduction to spoors [**21**].

A key matter in Mucormycosis management is the acknowledgment of the correct diagnosis [**11**]. Based on the results of the studies, the infection diagnosis by blood culture before death of neutropenia patients is unusual [**6**,**11**]. In addition, sputum culture was rarely useful in patients with pulmonary involvement. So, the diagnosis is possible by invading methods (biopsy or surgery for culture and histology) [**11**]. Unfortunately, using these methods is difficult and risky in most of the patients with hematologic malignancies especially those with pulmonary involvement due to their very bad general status and severe thrombocytopenia. For this reason, there is yet no standard method [**6**]. In the present study, we tried to investigate the clinical signs and experiments to have a more accurate diagnosis of the infection cases in order to diagnose the infection based on infection invasion to peripheral bones and soft tissues in CT scan and MRI where biopsy is impossible. Therefore, these problems in the clinical diagnosis of the infection are the main reasons for the lack of clear insight about the epidemiology of the infection and this issue caused most of the patients to be under presumptive therapies for Mucormycosis treatment and also many patients were not recognized because of the non-existence of standard proving method [**5**].

According to the results of studies, removing Mucormycosis infection may need several approaches simultaneously including anti-fungal treatment, surgery (debridement), and improving the predisposing condition [**6**,**24**]. In our study, just like in the study of Sarvestani et al., all the patients received Amphotericin B [**21**]. Treatment with Amphotericin B has several side effects especially in treating patients with nervous system Mucormycosis, these side effects, and penetration limitation causing a lack of effectiveness for this medicine. Therefore, replaced treatment of Liposomal Amphotericin B (L-AmB) was applied to treat the patients with a nervous system involvement in our study [**24**]. However, it is considerable that using this medicine has a limitation due to its high price and unavailability [**21**]. Also, like in the reports of some studies, our results showed that the treatment with Amphotericin B is not useful in most of the patients, while the use of a combination of this medicine and surgery (debridement) reduces death [**19**,**24**].

In spite of recent medical developments, this fungal infection has weak prognosis. Generally, 65.5% of death was reported in this study, that being in agreement with figures reported in the study done by Gleissner et al. and Liviopagani et al. [**3**,**24**]. According to the studies performed, the death rate is so much different and it has been observed to be between 33.3-63% in Korean and Italian studies and reached 96% in spread Mucormycosis patients [**1**,**4**,**24**]. These differences related to death caused by Mucormycosis may be accountable for many factors including the early diagnosis, infection place, and the person’s status of immune system, improving accompanied factors, and applied type of treatment [**21**].

In spite of limitations related to the retrospective type of the study, our study suggested valuable information. One advantage of the study is the collection of information of the hospitalized patients having hematologic malignancies during the study. Our study confirmed that among the patients of hematologic malignancies, patients with AML had the highest risk of generating the Mucormycosis infection and continuing intense chemotherapy increased infection risk by generating neutropenia patients. Death reduction related to Mucormycosis infection by combined therapy of using Amphotericin B and surgery (debridement) was the most notable finding of the study, which can cause an improvement in the prognosis of the patients having the infection. Since the Mucormycosis infection has a high mortality rate, preventing and reducing the incidence rate of these infections seems to be a priority for the patients with hematologic malignancies, in spite of developed therapies in best medical centers.

## Conclusion

According to the issues discussed earlier, the prevalence of this infection among patients having hematologic cancers is considerable. On the other hand, the prevalence of chronic diseases such as hematologic malignancies, that are the most important risk factors of the infection, is increasing. Therefore, expecting a further rise of the real prevalence rate for these diseases is not far from the mind compared to estimated rate of the present study. However, we can reduce the progress of the infection with controlling and preventing underlying diseases. Although we want to slow the progress, interventions and their effects are time consuming. Therefore, we cannot expect to reduce these rates even with very good and fundamental interventions. As a result, this issue highlighted that secondary prevention is of special importance as early diagnosis in place of primary prevention in this area. Assuring secondary prevention in all the patients hospitalized in the hospitals may not be acceptable and effective but it must be considered in the people in high risk, such as the neutropenia patients presenting hematologic malignancies, especially those with acute leukemic malignancy, so that incurred dangers can be minimized. Since the diagnosis time and treatment are very important, it is better to start the treatment of Mucormycosis disease in case there are suspicions regarding the clinical symptoms and no waiting is preferred for the biopsy result (culture and smear). Also because the most common way of the infection transfer is through the inhalation of spoors, taking the proper measures for controlling and changing the environment that reduce the risk of exposure to fungal spoors, seems the best approach for the prevention of the fatal infection.

**Recommendations**

Developing scientific researches and performing cohort studies to estimate the incidence rate of different hematologic malignancies and also risk factors of the infection.

Accurate recording and establishment of the electronic information bank for the infection in medical document centers, so that researchers could use them and inform policy makers and planners

Developing knowledge and mental presence of doctors regarding the symptoms of the disease in susceptible patients and regarding the diagnosis and quick treatment of the infection among these patients.

Warning patients about hematologic malignancies regarding the symptoms of Mucormycosis disease.

**Acknowledgement**

Last but not least, we would like to greatly acknowledge the financial supports for the research and technology assistance of the Medical Sciences University of Iranian Army, respectful personnel of Imam Reza Hospital, especially specialists and nurses of blood, medical documents, laboratory and pathology units for helping us in collecting the data.
